# Involvement of TLR4 in the protective effect of intra-articular administration of curcumin on rat experimental osteoarthritis^1^

**DOI:** 10.1590/s0102-865020190060000004

**Published:** 2019-08-19

**Authors:** Dan Yan, Bingshu He, Jie Guo, Shulan Li, Jun Wang

**Affiliations:** I Associate Professor, Hubei Province Key Laboratory of Occupational Hazard Identification and Control, Wuhan University of Science and Technology, China. Conception and design of the study, acquisition and interpretation of data.; II MS, Department of Orthopedics, Hubei Provincial Women and Children’s Hospital, China. Conception and design of the study, acquisition and interpretation of data.; III MS, Hubei Province Key Laboratory of Occupational Hazard Identification and Control, Wuhan University of Science and Technology, China. Acquisition of data, critical revision.; IV MS, Hubei Province Key Laboratory of Occupational Hazard Identification and Control, Wuhan University of Science and Technology, China. Acquisition of data.; V Associate Professor, Hubei Province Key Laboratory of Occupational Hazard Identification and Control, Wuhan University of Science and Technology, China. Design of the study, manuscript writing, critical revision, supervised all phases of the study.

**Keywords:** Osteoarthritis, Curcumin, Toll-Like Receptor 4, Lipopolysaccharides, Rats

## Abstract

**Purpose:**

In view of the principal role of Toll-like receptor 4 (TLR4) in mediating sterile inflammatory response contributing to osteoarthritis (OA) pathogenesis, we used lipopolysaccharide (LPS), a known TLR4 activator, to clarify whether modulation of TLR4 contributed to the protective actions of intra-articular administration of curcumin in a classical rat OA model surgically induced by anterior cruciate ligament transection (ACLT).

**Methods:**

The rats underwent ACLT and received 50μl of curcumin at the concentration of 1 mg mL^-1^ and 10 μg LPS by intra-articular injection once a week for 8 weeks. Morphological changes of the cartilage and synovial tissues were observed. Apoptotic chondrocytes were detected using TUNEL assay. The concentrations of IL-1β and TNF-ɑ in synovial fluid were determined using ELISA kits. The mRNA and protein expression levels of TLR4 and NF-κB p65 were detected by real-time PCR and Western blotting, respectively.

**Results:**

Intra-articular administration of curcumin significantly improved articular cartilage injury, suppressed synovial inflammation and down-regulated the overexpression of TLR4 and its downstream NF-κB caused by LPS-induced TLR4 activation in rat osteoarthritic knees.

**Conclusion:**

The data suggested that the inhibition of TLR4 signal might be an important mechanism underlying a protective effect of local curcumin administration on OA.

## Introduction

As the most active component in a popular spice known as turmeric with a strong safety record, curcumin has been considered to be a potential natural antiosteoarthritic agent under limelight^1,2^. In traditional Chinese and Indian medicine, turmeric that is the dried rhizome and grounded root of the plant *Curcuma longa* has long been used in patients suffering from inflammatory and degenerative disorders^1,3^. Composition analysis showed, in addition to other macro- and micronutrients, curcumin accounted for 77% of the extract of turmeric^1^, and the turmeric powder contained 5% curcumin^4^. Numerous *in vitro* studies^2,5-8^ have demonstrated the potential beneficial effects of curcumin in osteoarthritis (OA). Despite the encouraging findings these *in vitro* studies provide, an important barrier should be considered before the *in vivo* administration of curcumin in OA control. That is the very low bioavailability of naturally occurring curcumin^1,4^. Yang *et al.* ^9^ have reported that a single oral dosage of curcumin at 500 mg/kg in rat only resulted in a maximum plasma concentration of 0.06 μg/mL, which suggested an oral bioavailability of 1%. Similar findings were also observed in humans^10^. Therefore, as a patient-friendly and ideal drug delivery method commonly-used in OA therapies, intra-articular administration could result in a concentrated therapeutic dose of curcumin throughout the joint capsule, and increase the curcumin’s bioavailabilities at the disease site for OA treatment^10,11^.

So far, the mechanisms underlying the antiosteoarthritic activity of curcumin are still unclear. In recent years, the principal role of Toll-like receptor 4 (TLR4) in mediating sterile inflammatory response contributing to OA pathogenesis has been established^12-14^. TLR4 is the most studied subtype of TLRs, the latter of which are innate immune pattern recognition receptors having ability to elicit robust pro-inflammatory cytokine production upon ligand activation. The expression of TLR4 in joint tissues is augmented with increasing severity of OA^12^. And the activation of TLR4 by damage-associated molecules would further trigger chondrocyte- mediated inflammatory responses^13^. In a pilot study, TLR4 knockout mice receiving patellofemoral cartilage injury had been demonstrated improved gait and limited progression of OA compared to wild type controls^14^. And systemic TLR4 antagonism in a surgical murine OA model resulted in a significant decrease in gross and histopathologic changes^14^. Therefore, TLR4-specific disease-modifying OA drugs (DMOADs) offer considerable promise for the control of OA by inhibiting TLR4 responses locally in the joint^12,13^.

Up to date, the TLR4 inhibitory activity of curcumin has been demonstrated in liver cancer HepG2 cells^15^, experimental traumatic brain injury^16^, and apolipoprotein E-knockout mice^17^ *etc.*, which has been believed as a key underlying mechanism of curcumin’s anti-inflammatory properties. Here, we used lipopolysaccharide (LPS), a known TLR4 activator, to clarify whether modulation of TLR4 contributed to the protective actions of intra-articular administration of curcumin in a classical rat OA model surgically induced by anterior cruciate ligament transection (ACLT).

## Methods

### Animals and groups

The experiment protocols were approved by the Animals Care and Use Committee of Wuhan University of Science and Technology (Project number: WUST-18501).

Male SD rats, weighing 200-220 g, were obtained from Hubei Experimental Animal Research Center (Hubei, China). After a 1-week acclimation period, all animals were randomly divided into 5 groups of 10 rats in each group: (1) sham-operated control group; (2) OA model control group; (3) curcumin intra-articular administration group; (4) LPS intervention group and (5) LPS intervention + curcumin intra-articular administration group. The anterior cruciate ligaments in the right knees of rats in 2-5 groups were surgically transected as previously described^18-20^ to establish OA model. Arthrotomy without anterior cruciate ligament damage was performed in sham-operated rats. Four weeks after surgery, the rats in the group 3 and group 5 received 50μl of curcumin (Sigma Chemical Co.,St. Louis, MO, USA) at the concentration of 1 mg mL^-1^ by intra-articular injection once a week for 8 weeks. Meanwhile, 10 μg LPS was intra-articularly injected into the right knees of the rats in 4 and 5 groups once a week. The sham-operated and OA model groups received an injection of 50 μl saline into the knee joints as control. At 24 h after the last administration, all rats were sacrificed, synovial fluid and the right knee joints were collected.

### Histological analysis

The proximal tibias and synovial tissues were dissected from the collected knee joints, fixed with 10% neutral-buffered formalin, subsequently decalcified, dehydrated and embedded in paraffin. The cartilage and synovial tissues were stained with hematoxylin and eosin (HE) to observe the histopathological changes. Safranin O/Fast Green staining was used to assess the glycosaminoglycan content in cartilage tissue sections.

### TUNEL assay

The apoptotic chondrocytes in the sections were detected using the terminal deoxynucleotidyl transferase mediated dUTP nick end labelling (TUNEL) assay following the instruction manual of TUNEL Apoptosis Assay Kit (Servicebio, Wuhan, China) .

### Measurement of interleukin (IL)-1β and tumor necrosis factor (TNF)-ɑ levels in synovial fluid

Synovial fluid lavage of rats was collected from the knee joints immediately after sacrifice^20^. The concentrations of IL-1β and TNF-ɑ in synovial fluid were determined using ELISA kits according to the manufacturer’s instructions (R&D Systems, USA). The urea levels in synovial fluid lavage of each rat were measured using the QuantiChrom Urea Assay Kit(BioAssay Systems, Hayward, CA) to correct the IL-1β and TNF-ɑ values for dilution.

### Real-time PCR

Total RNA from articular cartilage and synovial tissues was extracted using a TRIzol Reagent. Then cDNA was generated using the All-in-OneTM First-Strand cDNA Synthesis kit (GeneCopoeia, USA). TLR4 primers: 5’- CGC TTT CAC CTC TGC CTT CAC TAC AG -3’(forward) and 5’- ACA CTA CCA CAA TAA CCT TCC GGC TC -3’(reverse); NF-κB p65 primers: 5’- GCT TTG CAA ACC TGG GAA TA -3’(forward) and 5’- TCC GCC TTC TGC TTG TAG AT -3’(reverse); β-actin primers: 5’- GAT TAC TGC TCT GGC TCC TAG C -3’(forward) and 5’- GAC TCA TCG TAC TCC TGC TTG C -3’(reverse). An Applied Biosystems StepOnePlus™ Real-Time PCR System was used to carry out the real-time PCR analysis. Levels of TLR4 and NF-κBp65 mRNA were normalized against that of β-actin mRNA in the same sample.

### Western blotting

The protein expression levels of TLR4 and NF-κBp65 were detected by Western blotting. Proteins in the cartilage and synovial tissues from rat knee joints were separated by SDS-PAGE, then transferred onto a nitrocellulose sheet. Membrane was blocked and incubated with a 1:1000 dilution of TLR4, NF-κBp65 and β-actin antibody (Santa Cruz, CA) for 2h. After incubation with the corresponding secondary antibody, proteins were visualized using an ECL chemiluminescence detection kit (Advansta, USA). The level of protein expression was corrected by that of β-actin in the same sample.

### Statistical analysis

All data were expressed as the mean ± SD, and analyzed using one-way analysis of variance (ANOVA) with post hoc Tukey test by SPSS 22.0 software. P<0.05 or P<0.01 was considered statistically significant.

## Results

### Intra-articular administration of curcumin improved articular cartilage injury caused by TLR4 activation in rat osteoarthritic knees

**Figure 1:**
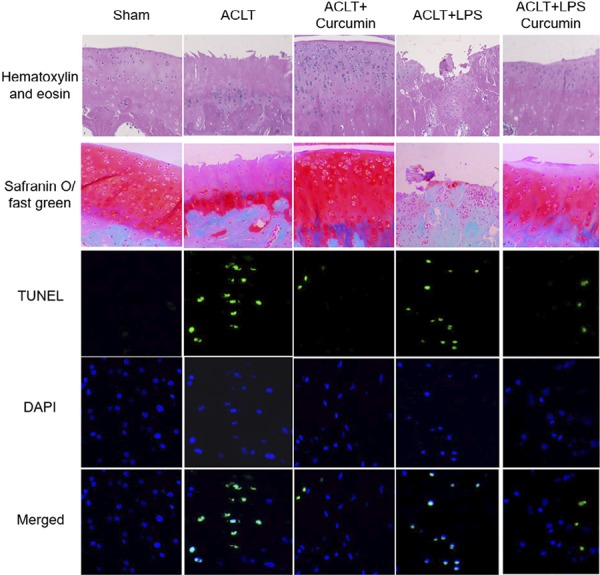
Representative photomicrographs of hematoxylin and eosin (HE) (200× magnification), Safranin O-fast green (200× magnification) and TUNEL (400× magnification) -stained cartilage tissue sections of rat tibial plateau.

The general morphology of cartilage was assessed using HE staining, and the cartilage proteoglycan content was determined by Safranin O/Fast Green staining (Fig. 1). Compared with ACLT-induced OA group, TLR4 activation induced by LPS injection into ACLT-operated rat osteoarthritic knees led to an obvious increase in the cartilage damage and articular cartilage thickness, the layer structure of cartilage was almost absent and more severe lesions were observed in the ACLT+LPS group. However, intra-articular administration of curcumin well preserved cartilage matrix integrity, retained the superficial layer and the structure of cartilage tissues, ameliorated cartilage lesion and matrix degradation induced by either ACLT or ACLT+LPS intervention.

Then, TUNEL staining was performed in cartilage specimens to detect chondrocyte apoptosis (Fig. 1). LPS-induction into rat OA knees decreased the total chondrocyte numbers, but increased the percentage of TUNEL-positive apoptotic chondrocytes, suggesting that TLR4 activation in OA knee joints might accentuate chondrocyte apoptosis. Importantly, intra-articular administration of curcumin reversed the apoptosis induction effect of TLR4 activation in OA.

### Intra-articular administration of curcumin suppressed synovial inflammation caused by TLR4 activation in rat osteoarthritic knees

**Figure 2:**
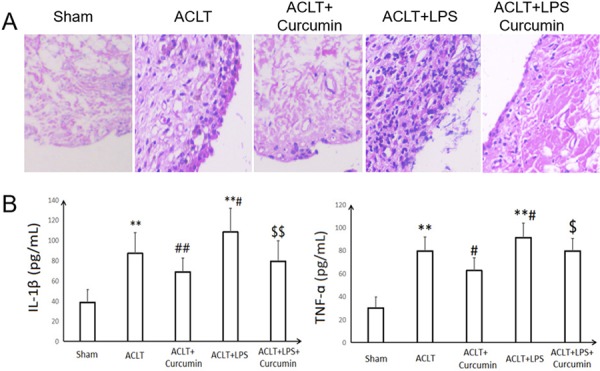
Effect of intra-articular administration of curcumin on synovial inflammation induced by LPS induction into rat osteoarthritic knee. (A) Representative images of HE -stained synovial tissues (200× magnification). (B) The concentrations of IL-1β and TNF-ɑ in synovial lavage. **P<0.01, compared with the sham control group; #P<0.05, ##P<0.01, compared with the ACLT OA model group; $ P<0.05, $$ P<0.01, compared with the ACLT+LPS group.

As a result of interactions between the joint damage factors and immune system, synovial inflammation is a hallmark feature of knee OA, and plays a critical role in the OA pathological process^21,22^. Accordingly, anti-inflammatory therapeutics offer new opportunities for OA control^23^. Here, synovial inflammation was measured by HE staining on synovial tissue sections, and ELISA assay in the synovial fluid lavage. As shown in Figure 2, after stimulation by TLR4 activator LPS, the synovial inflammation induced by OA was further accentuated. Importantly, HE staining in the synovial membrane showed reduced inflammatory cell infiltration and less synovial lining cell layer in curcumin-administrated rats compared with ACLT or ACLT+LPS groups.

The robust production of two important inflammatory cytokines interleukin (IL)-1β and tumor necrosis factor (TNF)-ɑ following TLR4 activation contributes to the induction of joint inflammation and cartilage catabolism in OA^13,23,24^. We found that the urea-adjusted synovial lavage concentrations of IL-1β and TNF-ɑ were significantly enhanced in ACLT+LPS groups, compared to the ACLT OA group (P<0.05). However, the IL-1β and TNF-ɑ contents in the rat synovial lavage were markedly decreased after local curcumin administration in OA or LPS-stimulated OA rats (P<0.05; P<0.01). Intra-articular administration of curcumin suppressed LPS-induced IL-1β and TNF-ɑ secretion from synovium in OA rats by 26.8 and 12.6%, respectively.

### Intra-articular administration of curcumin down-regulated LPS-induced overexpression of TLR4 and its downstream NF-κB in OA knee joints

**Figure 3:**
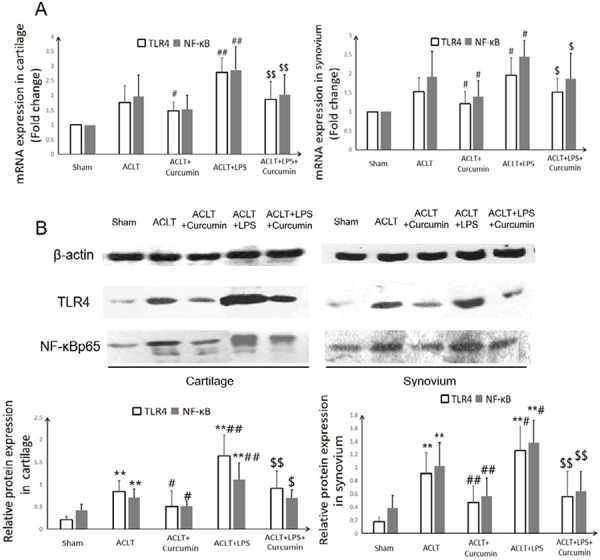
- Effect of intra-articular administration of curcumin on the mRNA (A) and protein (B) expressions of TLR4 and its downstream NF-κB in cartilage and synovial tissues of OA knee joints.**P<0.01, compared with the sham control group; # P<0.05, ##P<0.01, compared with the ACLT OA model group; $ P<0.05, $$ P<0.01, compared with the ACLT+LPS group.

As shown in Figure 3, the overexpression of TLR4 in mRNA and protein level was found in both cartilage and synovium tissues from LPS-administrated rat OA joints (P<0.01), which was consistent with the classical role of LPS as TLR4 agonist. However, the increase in TLR4 expression induced by LPS was attenuated after intra-articular administration of curcumin (P<0.05; P<0.01).

In response to TLR4 ligand activation, nuclear factor (NF)-κB signaling is induced and involved in the development and progression of OA^13^. As a key mediator of TLR4 activation and subsequent inductive effects on inflammatory cytokines IL-1β and TNF-ɑ, NF-κB plays a distinctive role in OA pathogenesis through affecting chondrocyte apoptosis, synovial inflammation, cartilage matrix remodeling, and stimulating the downstream regulators of terminal chondrocyte differentiation^25^. In line with the changes in the TLR4 expression, local administration of curcumin in knee joints markedly repressed the LPS-induced overexpression of NF-κB (P<0.05; P<0.01).

## Discussion

During the last decades, the key role of inflammation has been well-accepted in the OA pathogenesis. Local inflammation not only is present in most OA patients, but also, actively serves as the initiator in the progression of this disease^1,13,22,26^. Influx of inflammatory cells has been observed

in the cartilage and synovium, and a plethora of inflammatory mediators have been demonstrated present in tissues and fluids from OA patients. These pathological inflammatory processes further cause the production of degrading enzymes which break down the cartilage extracellular matrix. And the subsequent release of damage associated molecular patterns from the breakdown of cartilage matrix contributes a vicious cycle of OA progression linked to inflammation^22,26^. Accordingly, the use of anti-inflammatory agents has been considered as the cornerstone of OA management^1,22,26^. Here we confirmed the anti-inflammatory role of curcumin in OA animal models, which was in line with the previous *in vitro* studies^2,5-8^. In the present study, local curcumin administration in OA rats significantly improved the pathological inflammatory lesions in cartilage and synovial membrane, decreased the concentrations of inflammatory cytokines IL-1β and TNF-ɑ in synovial lavage.

Most importantly, inflammation and inflammation-induced catabolism (e.g. activation of matrix metalloproteinases) in OA are tightly controlled by TLR-mediated innate immune responses^13^. In particular, the high expression of TLR4 by chondrocytes, synoviocytes, osteoblasts and immune cells supports its role in the physiology and pathology of joint tissues. Throughout the development of OA, TLR4 expression in the lesional areas of joints is increased. And activation of TLR4 would trigger inflammatory and catabolic responses by chondrocytes, thus is closely involved in OA-related cartilage damage^13^. The main aim of our study was to determine whether the TLR4 inhibitory activity of curcumin, which has been demonstrated in other pathological conditions such as liver cancer^15^, traumatic brain injury^16^ and atherosclerosis^17^, contributed to its protective anti-inflammatory effect of curcumin on OA. And LPS, a canonical TLR4 activator, was used as a tool to up-regulate TLR4 in the joints. The results showed that local administration of curcumin in knee joints markedly repressed the LPS-induced IL-1β and TNF-ɑ secretion from synovium, inhibited the LPS-induced overexpression of TLR4 and its downstream NF-κB in cartilage and synovium tissues, ameliorated the LPS-aggravated cartilage lesion, matrix degradation, chondrocyte apoptosis and synovial inflammation. These data suggested that, curcumin exerted its anti-inflammatory effect on OA, at least in part, through TLR4 pathway.

## Conclusions

As a conclusion, this study demonstrated the antiosteoarthritic effect of intra-articular administration of curcumin on a rat OA model surgically induced by ACLT, most importantly, intra-articular administration of curcumin in LPS-injected rat osteoarthritic knees significantly ameliorated the histological lesion and matrix degradation in articular cartilage caused by LPS-induced TLR4 activation, improved the LPS-stimulated chondrocyte apoptosis and inflammatory cell infiltration in synovial membrane, decreased the enhanced IL-1β and TNF-ɑ contents in synovial lavage, and down-regulated LPS-induced overexpression of TLR4 and its downstream NF-κB in cartilage and synovium tissues. These findings suggested the involvement of TLR4 in the protective effect of local administration of curcumin on OA, which might be an important mechanism underlying the attractive potential of curcumin in OA control.

## Footnotes

1 Research performed at Hubei Province Key Laboratory of Occupational Hazard Identification and Control, Wuhan University of Science and Technology, China.
